# The German National Pandemic Cohort Network (NAPKON): rationale, study design and baseline characteristics

**DOI:** 10.1007/s10654-022-00896-z

**Published:** 2022-07-29

**Authors:** Maximilian Schons, Lisa Pilgram, Jens-Peter Reese, Melanie Stecher, Gabriele Anton, Katharina S. Appel, Thomas Bahmer, Alexander Bartschke, Carla Bellinghausen, Inga Bernemann, Markus Brechtel, Folke Brinkmann, Clara Brünn, Christine Dhillon, Cornelia Fiessler, Ramsia Geisler, Eckard Hamelmann, Stefan Hansch, Frank Hanses, Sabine Hanß, Susanne Herold, Ralf Heyder, Anna-Lena Hofmann, Sina M. Pütz, Anna Horn, Carolin Jakob, Steffi Jiru-Hillmann, Thomas Keil, Yascha Khodamoradi, Mirjam Kohls, Monika Kraus, Dagmar Krefting, Sonja Kunze, Florian Kurth, Wolfgang Lieb, Lena Johanna Lippert, Roberto Lorbeer, Bettina Lorenz-Depiereux, Corina Maetzler, Olga Miljukov, Matthias Nauck, Daniel Pape, Valentina Püntmann, Lennart Reinke, Christoph Römmele, Stefanie Rudolph, Julian Sass, Christian Schäfer, Jens Schaller, Mario Schattschneider, Christian Scheer, Margarete Scherer, Sein Schmidt, Julia Schmidt, Kristina Seibel, Dana Stahl, Fridolin Steinbeis, Stefan Störk, Maike Tauchert, Johannes Josef Tebbe, Charlotte Thibeault, Nicole Toepfner, Kathrin Ungethüm, Istvan Vadasz, Heike Valentin, Silke Wiedmann, Thomas Zoller, Eike Nagel, Michael Krawczak, Christof von Kalle, Thomas Illig, Stefan Schreiber, Martin Witzenrath, Peter Heuschmann, Jörg Janne Vehreschild, NAPKON Research Group

**Affiliations:** 1 Department I of Internal Medicine, Center for Integrated Oncology Aachen Bonn Cologne Duesseldorf, Faculty of Medicine and University Hospital Cologne, University of Cologne, Cologne, Germany; 2 Department II of Internal Medicine, Hematology/Oncology, Goethe University, Frankfurt, Germany; 3 Institute of Clinical Epidemiology and Biometry, University of Würzburg, Würzburg, Germany; 4 German Center for Infection Research (DZIF), Partner-Site Cologne-Bonn, Cologne, Germany; 5 Institute of Epidemiology, Helmholtz Center Munich, Munich, Germany; 6 German Centre for Infection Research (DZIF), Partner Site Munich, Munich, Germany; 7 Internal Medicine Department I, University Hospital Schleswig-Holstein, Campus Kiel, Kiel, Germany; 8 Airway Research Center North (ARCN), German Center for Lung Research (DZL), Grosshansdorf, Germany; 9 Core Facility Digital Medicine and Interoperability, Berlin Institute of Health at Charité – Universitätsmedizin Berlin, Berlin, Germany; 10 Department of Respiratory Medicine and Allergology, Medical Clinic 1, University Hospital Frankfurt, Goethe University, Frankfurt, Germany; 11 Hannover Medical School, Hannover Unified Biobank, Hannover, Germany; 12 Department of Paediatric Pneumology, Allergy and CF- Centre, University Children’s Hospital, Ruhr- University Bochum, Bochum, Germany; 13 COVID-19 Task Force, University Hospital Augsburg, Augsburg, Germany; 14 Department of Pediatrics, Children’s Center Bethel, University Hospital East Westphalia, University Bielefeld, Bielefeld, Germany; 15 Department for Infectious Diseases and Infection Control, University Hospital Regensburg, Regensburg, Germany; 16 Emergency Department, University Hospital Regensburg, Regensburg, Germany; 17 University Medical Center Göttingen (UMG), Göttingen, Germany; 18 German Center for Cardiovascular Diseases (DZHK), Berlin, Germany; 19 Department of Internal Medicine V, University Hospital Giessen and Marburg, Justus-Liebig-University Giessen, Giessen, Germany; 20 Department of Internal Medicine, German Center for Lung Research (DZL), Universities of Giessen and Marburg Lung Center (UGMLC), Justus Liebig University Giessen, Giessen, Germany; 21 Institute for Lung Health (ILH), Giessen, Germany; 22 NUM Coordination Office, Charité – Universitätsmedizin Berlin, Charitéplatz 1, 10117 Berlin, Germany; 23 Insitute of Clinical Epidemiology and Biometry, University of Würzburg, Würzburg, Germany; 24 Institute of Social Medicine, Epidemiology and Health Economics, Charité - Universitätsmedizin Berlin, Berlin, Germany; 25 State Institute of Health, Bavarian Health and Food Safety Authority, Bad Kissingen, Germany; 26 Department of Infectious Diseases, Medical Clinic 2, University Hospital Frankfurt, Goethe University Frankfurt, Frankfurt, Germany; 27 German Center for Cardiovascular Research (DZHK), Partner Site Munich, Munich, Germany; 28 Department of Infectious Diseases and Respiratory Medicine, Charité – Universitätsmedizin Berlin, Freie Universität Berlin and Humboldt-Universität zu Berlin, Charitéplatz 1, 10117 Berlin, Germany; 29 Department of Tropical Medicine, Bernhard Nocht Institute for Tropical Medicine, and Department of Medicine I, University Medical Centre Hamburg-Eppendorf, Hamburg, Germany; 30 Institute of Epidemiology, Kiel University, Kiel, Germany; 31 Department of Radiology, University Hospital, LMU, Munich, Germany; 32 Medical Heart Center of Charité and German Heart Institute Berlin, Institute of Computer-Assisted Cardiovascular Medicine, Berlin, Germany; 33 Department of Neurology, University Hospital Schleswig-Holstein, Campus Kiel, Kiel University, Kiel, Germany; 34 Institute of Clinical Chemistry and Laboratory Medicine, University Medicine Greifswald, Greifswald, Germany; 35 DZHK (German Center for Cardiovascular Research), Partner Site Greifswald, Greifswald, Germany; 36 Department I of Internal Medicine, University Hospital Schleswig-Holstein, Campus Kiel, Kiel, Germany; 37 Institute for Experimental and Translational Cardiovascular Imaging, University Hospital Frankfurt am Main, Frankfurt, Germany; 38 Department of Internal Medicine I, University Hospital Schleswig-Holstein, Campus Kiel, Kiel, Germany; 39 Charité – Universitätsmedizin Berlin, Freie Universität Berlin and Humboldt Universität zu Berlin, Berlin Institute of Health (BIH) at Charité – Universitätsmedizin Berlin, Joint Charité and BIH Clinical Study Center, Charitéplatz 1, 10117 Berlin, Germany; 40 Institute of Clinical Chemistry and Laboratory Medicine, University Medicine Greifswald, Greifswald, Germany; 41 DZHK e.V. (German Centre for Cardiovascular Research), Partner Site Greifswald, Greifswald, Germany; 42 Charité – Universitätsmedizin Berlin, Freie Universität Berlin and Humboldt – Universität zu Berlin, Charitéplatz 1, 10117 Berlin, Germany; 43 DZHK (German Centre for Cardiovascular Research), Partner Site Berlin, Berlin, Germany; 44 Department of Anesthesiology and Intensive Care Medicine, University Medicine Greifswald, Greifswald, Germany; 45 Berlin Institute of Health at Charité – Universitätsmedizin Berlin, Clinical Study Center, Charitéplatz 1, 10117 Berlin, Germany; 46 University Medicine Greifswald, Greifswald, Germany; 47 Comprehensive Heart Failure Center, University and University Hospital Würzburg, Würzburg, Germany; 48 Department of Internal Medicine I, University Hospital Würzburg, Würzburg, Germany; 49 Department of Gastroenterology and Infectious Disease, University Medical Center East Westphalia-Lippe, Klinikum Lippe, Detmold, Germany; 50 Department of Pediatrics, Carl Gustav Carus University Hospital, TU Dresden, Dresden, Germany; 51 Department of Internal Medicine, University Hospital Giessen and Marburg, Justus Liebig University Giessen, Giessen, Germany; 52 Universities of Giessen and Marburg Lung Center (UGMLC), German Center for Lung Research (DZL), Frankfurt, Germany; 53 Institute for Experimental and Translational Cardiovascular Imaging, University Hospital Frankfurt am Main, Frankfurt, Germany; 54 Institute of Medical Informatics and Statistics, Kiel University, University Hospital Schleswig-Holstein, Kiel, Germany; 55 Department of Internal Medicine I, University Hospital Schleswig Holstein, Kiel University, Kiel, Germany; 56 Department of Infectious Diseases and Respiratory Medicine, Charité - Universitätsmedizin Berlin, Charitéplatz 1, 10117 Berlin, Germany; 57 German Center for Lung Research (DZL), Frankfurt, Germany; 58 Clinical Trial Center Würzburg, University Hospital Würzburg, Würzburg, Germany; 59 Department of Internal Medicine, Hematology/Oncology, Goethe University, Frankfurt,, Germany; 60 German Centre for Infection Research (DZIF), Partner Site Bonn-Cologne, Cologne, Germany

**Keywords:** COVID-19, SARS-CoV-2, Prospective national cohort, Longitudinal study, Epidemiology, Cross-sectoral, Population-based

## Abstract

The German government initiated the *Network University Medicine* (NUM) in early 2020 to improve national research activities on the *Severe Acute Respiratory Syndrome Coronavirus 2* (SARS-CoV-2) pandemic. To this end, 36 German Academic Medical Centers started to collaborate on 13 projects, with the largest being the *National Pandemic Cohort Network* (NAPKON). The NAPKON’s goal is creating the most comprehensive *Coronavirus Disease 2019* (COVID-19) cohort in Germany. Within NAPKON, adult and pediatric patients are observed in three complementary cohort platforms (Cross-Sectoral, High-Resolution and Population-Based) from the initial infection until up to three years of follow-up. Study procedures comprise comprehensive clinical and imaging diagnostics, quality-of-life assessment, patient-reported outcomes and biosampling. The three cohort platforms build on four infrastructure core units (Interaction, Biosampling, Epidemiology, and Integration) and collaborations with NUM projects. Key components of the data capture, regulatory, and data privacy are based on the *German Centre for Cardiovascular Research.* By April 01, 2022, 34 university and 40 non-university hospitals have enrolled 5298 patients with local data quality reviews performed on 4727 (89%). 47% were female, the median age was 52 (IQR 36–62-) and 50 pediatric cases were included. 44% of patients were hospitalized, 15% admitted to an intensive care unit, and 12% of patients deceased while enrolled. 8845 visits with biosampling in 4349 patients were conducted by April 03, 2022. In this overview article, we summarize NAPKON’s design, relevant milestones including first study population characteristics, and outline the potential of NAPKON for German and international research activities.

*Trial registration* https://clinicaltrials.gov/ct2/show/NCT04768998. https://clinicaltrials.gov/ct2/show/NCT04747366. https://clinicaltrials.gov/ct2/show/NCT04679584

**Supplementary Information:**

The online version contains supplementary material available at 10.1007/s10654-022-00896-z.

## Introduction

The pathogen *Severe Acute Respiratory Syndrome Coronavirus 2* (SARS-CoV-2) started to spread at the end of 2019 [1, 2], initiating the *Coronavirus Disease 2019* (COVID-19) pandemic [3]. As foreshadowed by the 2019 *Global Health Security* (GHS) report, no country was “fully prepared for epidemics or pandemics, and every country has important gaps to address” [4]. More than two years into the pandemic, by April 01, 2022, the *World Health Organization* (WHO) reported 485 million positive cases, and over 6.1 million deaths worldwide [5]. For Germany, the *Robert Koch Institute* (RKI) reported over 21.4 million positive cases and close to 130,000 deaths (as of April 01, 2022) [6].

In its pandemic preparedness program checklist, the WHO dedicates an entire section to “Research and Development”, listing essential and desirable activities for countries. Such activities include the development of study protocols, documentation of the evolution of epidemiological/clinical features, and outbreak investigations [7]. The RKI states similarly that “studies should be planned and prepared in advance of the pandemic so that they can be conducted rapidly at any time” [8]. However, Germany’s latest national pandemic plan from 2017 does not address specific research activities and contains no plans for studies of respective patient collectives [9]. Given the first signs of a pandemic in early 2020, the German *Federal Ministry of Education and Research* (BMBF) focused on the national establishment and streamlining of COVID-19 related scientific activities. As a result, the BMBF founded the *Network University Medicine* (NUM) in late March 2020 [10] to coordinate national research activities on SARS-CoV-2/COVID-19 and ensure pandemic preparedness of German academic medical centers in the future. The NUM has thus initiated joint SARS-CoV-2/COVID-19 research activities via 13 complementary projects by leveraging and connecting elements of existing academic research infrastructure in Germany, including all 36 university hospitals and additional collaborating (non-university) health care institutions [11]. The BMBF plans to continue the funding for NUM until 2024 [12].

The NUM’s largest project, the *National Pandemic Cohort Network* (NAPKON), is aiming to establish a standardized, high-quality data and biosample collection on patients, citizens, and controls with comparator respiratory infections. Next to international activities such as *International Severe Acute Respiratory and emerging Infection Consortium* (ISARIC) [13], many nations set up COVID-19 cohorts throughout 2020 across the globe [14–24].The NAPKON was initiated in July 2020 as Germany’s most comprehensive COVID-19 cohort. It was delineated from and aligned with three complementary German cohorts: the two already existing *Lean European Open Survey on SARS-CoV‑2 infected patients* (LEOSS) [25] and the Berlin prospective COVID-19 patient cohort (Pa-COVID-19) [26], as well as the proposal for the Post-COVID-Syndrome Study (COVIDOM) [27].

Here we report in detail on the NAPKON’s objectives, structures, and design. We present relevant milestones achieved and outline the potential of the NAPKON for German and international research activities.

## Methods

### Study design

The primary aim of the NAPKON is to create a harmonized, expandable, and interoperable network to support both the fight against the current COVID-19 pandemic as well as future pandemics of any origin.

The NAPKON consists of three parallel and complementary prospective cohort platforms that collect data and biosamples of SARS-CoV-2 infected patients, citizens, as well as controls with comparator respiratory infections during the acute phase and follow-up. Objectives for the usage of data and biosamples are to:

- Investigate frequency, severity and distinct phenotypes of COVID-19 and Post-COVID-19 Syndrome (PCS) in the German population and identify long-term clinical trajectories of PCS. POP
- Identify genomic, epigenomic, transcriptomic, proteomic, and metabolomic signatures predicting course and outcome of acute and post-acute COVID-19.
- Decipher further central pathophysiologic mechanisms of specific COVID-19 related pathologies in order to inform development of therapies.
- Establish commonalities and differences between COVID-19 and other forms of respiratory tract infections, pneumonia and acute respiratory distress syndrome (ARDS) in detail.
- Understand reasons for development of acute or post-acute COVID-19 in SARS-CoV- 2 vaccinated patients.

#### Each of the three cohort platforms focuses on one or multiple of the above scientific areas of interest. The following paragraphs will introduce the three cohort platforms and supporting infrastructure elements. Cross-sectoral platform

The Cross-Sectoral Platform (*Sektorenübergreifende Plattform*, SUEP) cohort recruits SARS-CoV-2 infected in- and outpatients of all ages across all departments and performs a comprehensive collection of primary health record data, basic clinical phenotyping (e.g., echocardiography, spirometry with full-body plethysmography) with biosample collection, and patient interviews/patient-reported outcome measures (PROM) (see Tables 1, 2) across all levels of health care providing facilities. This ensures cross-sectoral patient acquisition in the NAPKON. All German university and non-university hospitals and primary care practices can become study sites. In addition, mobile hotspot study teams are planned to cover long-term care and rehabilitation facilities. The cohort is registered at www.clinicaltrials.gov under NCT04768998.

**Table 1:** Overview of data collected within NAPKON by cohort platform

Category	Features	Collected in		
		SUEP	HAP	POP
Socio-demographic data	Age, sex, residence, marital status	**x**	**x**	**x**
	Educational level and employment status (e.g. general education degree, vocational degree)	**x**	**x**	**x**
Clinical data: pre-infection anamnestic data	Pre-infection lifestyle (e.g. sports activity, dietary pattern)	**x**		**x**
	Pre-infection smoking and alcohol consumption	**x**	**x**	**x**
	Pre-infection health status and functionality (e.g. Barthel Index, care level, Clinical Frailty Scale)	**x**	**x**	**x**
	Pre-infection medication	**x**	**x**	
	Vaccination status	**x**	**x**	**x**
	Comorbidities	**x**	**x**	**x**
	Directives for medical decisions (e.g. power of attorney, patient decree)	**x**	**x**	
Clinical data: parameters in the observational period	Infrastructural treatment context (e.g. health care facility, involved disciplines)	**x**	**x**	**x**
	Smoking and alcohol consumption	**x**		**x**
	Health status and functionality (e.g. Barthel index, care level, Clinical Frailty Scale)	**x**	**x**	**x**
	Symptoms, events	**x**	**x**	**x**
	Clinically indicated diagnostics (vital signs, pulmonary diagnostics, laboratory parameters, microbiology & virology, radiological findings, functional diagnostics)	**x**	**x**	**x**
	Intensive care scores (e. g. SOFA, SAPS)	**x**	**x**	
	Therapeutic measures (medication, interventions, surgery, complementary medicine)	**x**	**x**	**x**
	Pediatric-specific variable extensions (e.g. perinatal medical history, congenital defects, effects on development)	**x**		
Imaging data	Clinically indicated diagnostic imaging data	**x**	**x**	
	Study-related MRI scans		**x**	**x**
	Study related CT-Thorax scans		**x**	
	Study-related echocardiographies	**x**	**x**	**x**
Patient-reported outcome measures (PROM)	Cognitive function (e.g. PROMIS Kognition)	**x**	**x**	**x**
	Dypsnea (e.g. Modified Medical Research Council Dyspnea Scale, PROMIS Dyspnoe)	**x**	**x**	**x**
	Fatigue (e.g. Chalder Fatigue Scale, FACIT-F)	**x**	**x**	**x**
	Functional physical status (e.g. Activities of Daily Living)	**x**	**x**	**x**
	Mental health (e.g. GAD-7, Brief Resilience Scale)	**x**	**x**	**x**
	Pain (e.g. DN2, HIT-6)	**x**	**x**	**x**
	Quality of life (e.g. EQ-5D-5L)	**x**	**x**	**x**
Metadata	Study-related metadata (e.g. data quality assessment, protocol deviation)	**x**	**x**	**x**
	Digital Imaging and Communications in Medicine (DICOM) header information	**x**	**x**	**x**
	Biosample accompanying metadata (e.g. regarding transport, processing and storage)	**x**	**x**	**x**

**Table 2:** Overview of additional study assessments

Study assessment^a^	SUEP	HAP	POP
Abdominal ultrasonography			x
Additional medical history and recording by study physician	x	x	x
Basic endocrinological diagnostics		x	x
Computer tomography chest		x	
Electrocardiography	x	x	x
Electroencephalography		x	
Fraction Exspiratory Nitric Oxide			x
Fundus examination		x	
Home visit	x		
Impulse oscillometry			x
Long-term ECG		x	
Long-term glucose measurement		x	
Long-term RR		x	
Magnetic resonance imaging brain		x	x
Magnetic resonance imaging heart		x	
Microbiome sampling	x	x	x
Myocarditis panel		x	x
Basic neurological examination		x	x
6-Min walking test			x
Smell test		x	x
Spiroergometry		x	
Standard laboratory outpatients	x		
Standardized spirometry with bodyplethysmography and diffusion capacity	x	x	x
Taste test		x	x
Transthoracic echocardiography	x	x	x
Vital sign monitoring	x	x	x

^a^Modified for patients age < 18.

SUEP, cross-sectoral platform; HAP, high-resolution platform; POP, population-based platform

#### High-resolution platform

The High-Resolution Platform (*Hochauflösende Plattform*, HAP) cohort focuses on adult SARS-CoV-2 positive inpatients, especially those with a severe course of COVID-19, i.e., in need of intensive care unit treatment. Within the HAP, data and biosample collection are extended by a multidisciplinary study program of additional clinical examinations, supplementary cytokine profiling, and standardized imaging. The longitudinal biosample collection is of a much higher frequency as compared to the other two cohorts (see Tables 1, 2). The HAP cohort is conducted at ten German university hospitals with an adequate infrastructure for deep phenotyping. The cohort is registered at www.clinicaltrials.gov under NCT04747366.

#### Population-based platform

The Population-Based Platform (*Populationsbasierte Plattform*, POP) cohort focuses on describing health consequences of SARS-CoV-2 infection in the general adult population. It is conducted at established epidemiological centers at three university hospitals. Recruitment bias is minimized by contacting a stratified sample of SARS-CoV-2 positive individuals in three locally and structurally distinct German regions. Individuals are identified and contacted via local health authorities that are mandated to register all SARS-CoV-2 infections in their administrative districts. After consenting eligible individuals undergo a telephone interview and are subsequently invited for a baseline visit in the study center and yearly follow-ups. Visits include comprehensive clinical and functional health assessment in distinct organ systems, further interviews/PROM assessment, and biosample collection (see Tables 1, 2). The POP is registered at the *Deutsches Register Klinischer Studien* (DRKS) under DRKS00023742.

#### Infrastructure elements of the NAPKON

The NAPKON cohorts rest on a harmonized shared infrastructure provided by four NAPKON core units:

- The Interaction Core Unit (ICU) coordinates overall governance, support of the use & access processes, development of datasets, engagement of the scientific community via working parties and a scientific council, age-specific consideration of study aspects via a dedicated Pediatric Core Unit, convening of the general assembly, and other tasks related to internal project management or communication.
- The Biosample Core Unit (BCU) selects suitable biosamples together with clinical experts, defines standards of procedure for sampling, processing, storage, quality assurance, as well as regular auditing of biobanks.
- The Epidemiology Core Unit (ECU) is responsible for methodological consultation of the project and third parties applying for data/biosamples. It performs an external quality assurance and reporting of collected project data.
- The Integration Core Unit (IGCU) designs and manages the integration of external and existing cohort data into the NAPKON.

Since knowledge on COVID-19 was evolving quickly at the time the NAPKON was initiated, it was of highest priority to not only collect a comprehensive set of data and biosamples, but also to be able to change protocols and strategies on the move. This included the option to recontact patients and obtain consent for new and/or additional study interventions, e.g., inclusion in substudies and follow-up of children and adolescents after they attain full age. Central data storage and linkage of data from different sources requires a complex setup with trusted third-party and extensive communication and approval by data protection officers. Such a system was provided by the *German Centre for Cardiovascular Research* (*Deutsches Zentrum für Herz-Kreislauf-Forschung*, DZHK), including the following components [28]:

- *Clinical data management* electronic case report form (eCRF) for documentation of all clinical data, including additional tests performed as part of the study.
- *Imaging data management* central storage for clinical routine images and additional images collected by study protocol.
- *Biosample management* central laboratory information and management system (cLIMS).
- *Ethics coordination* coordination of ethical aspects regarding data-infrastructure and governance and professional communication with ethics committees and institutional review boards.
- *Trusted third party* centralized quality assurance and management of (electronic) informed consents, including supporting the invitation of patients to follow-up studies.
- *Data transfer office* provision of datasets and identification of the respective biosamples based on applications approved by the Use & Access committee.

### Study population and recruitment

Besides informed consent, the shared inclusion criterion (for primary cases—controls see below) of all three cohorts is a SARS-CoV-2-positive polymerase chain reaction (PCR) of a swab or body fluid.Alternatively, a negative molecular test with a very high clinical suspicion for a SARS-CoV-2 infection is regarded as a positive case (see Table 3 for details). Besides age < 18 for the POP and the HAP no exclusion criteria exist. The SUEP and HAP recruit patients within one week of meeting the inclusion criteria, the POP within 6–12 months after positive testing.

**Table 3:** Case and control definitions for the NAPKON.

Case definition for SARS-CoV-2 infection (= inclusion criteria)	Control definition
Either: Positive polymerase chain reaction (PCR) for SARS-CoV-2 in either Oro/nasopharyngeal swab, BAL, sputum, tracheal secretions, stool, or blood^a^ Or (all of the following): Negative polymerase chain reaction (PCR) for SARS-CoV-2 of a swab or body fluid Definitive infection of the respiratory system Characteristic radiographic imagery A negative test for influenza Exclusion of other potential causes (like chronic diseases of the respiratory system)	Case definition for SARS-CoV-2 case not applicable Applicable control inclusion criteria for one of the three control strata (pool) Outpatient (e.g., respiratory viral infection) Inpatient (e.g., community-acquired pneumonia) Intensive care unit (e.g., acute respiratory distress syndrome) Capacity for control recruitment with sufficient positive cases in the respective pool over the past eight weeks

Additional case definitions exist for patients age < 18. No exclusion criteria exist, except for age < 18 for the POP and the HAP

^a^Antibody testing or rapid tests are no viable alternatives

In total, the NAPKON aims to prospectively recruit and follow 7000 individuals (patients and controls) in the years 2020–2024, starting in November 2020. This sample size is delineated from allocating available funding evenly across high quality data and biosample acquisition in all disease strata (see Suppl Table S1 in the supplements). Study sites include all interested German university hospitals (SUEP & HAP, about 40% of the total study population), non-university hospitals, primary care practices, long-term care/rehabilitation facilities (SUEP, about 15% of the total study population), and patients/citizens contacted via local health authorities in three catchment areas (POP, about 45% of the total study population). Strata for various disease severities exist (see Suppl Table S1) and the recruitment for each cohort is balanced for disease severity. Additional stratification criteria for age, sex, infection date, vaccination status apply depending on the cohort. Given their capacities, the participating study sites are asked to consecutively enroll all eligible patients into the NAPKON. In addition to German, electronic and paper consent forms exist in eight different languages (e.g., English, Arabic, Turkish and French). Dedicated delegation procedures for patients who are incapable of giving consent (e.g., pediatric or unconscious patients) are available at most sites, further reducing selection bias.

A key asset of the NAPKON is the inclusion of 20% controls based on the pool recruitment method [29, 30]. The initial approach to compare COVID-19 with influenza proved not feasible due to very low infection rates by influenza virus in the 2020/21 season. To match comparator conditions effectively with different levels of severity of COVID-19, we therefore defined three different strata of controls (see Table 3).

### Visit schedules and follow-up

**Fig. 1:**
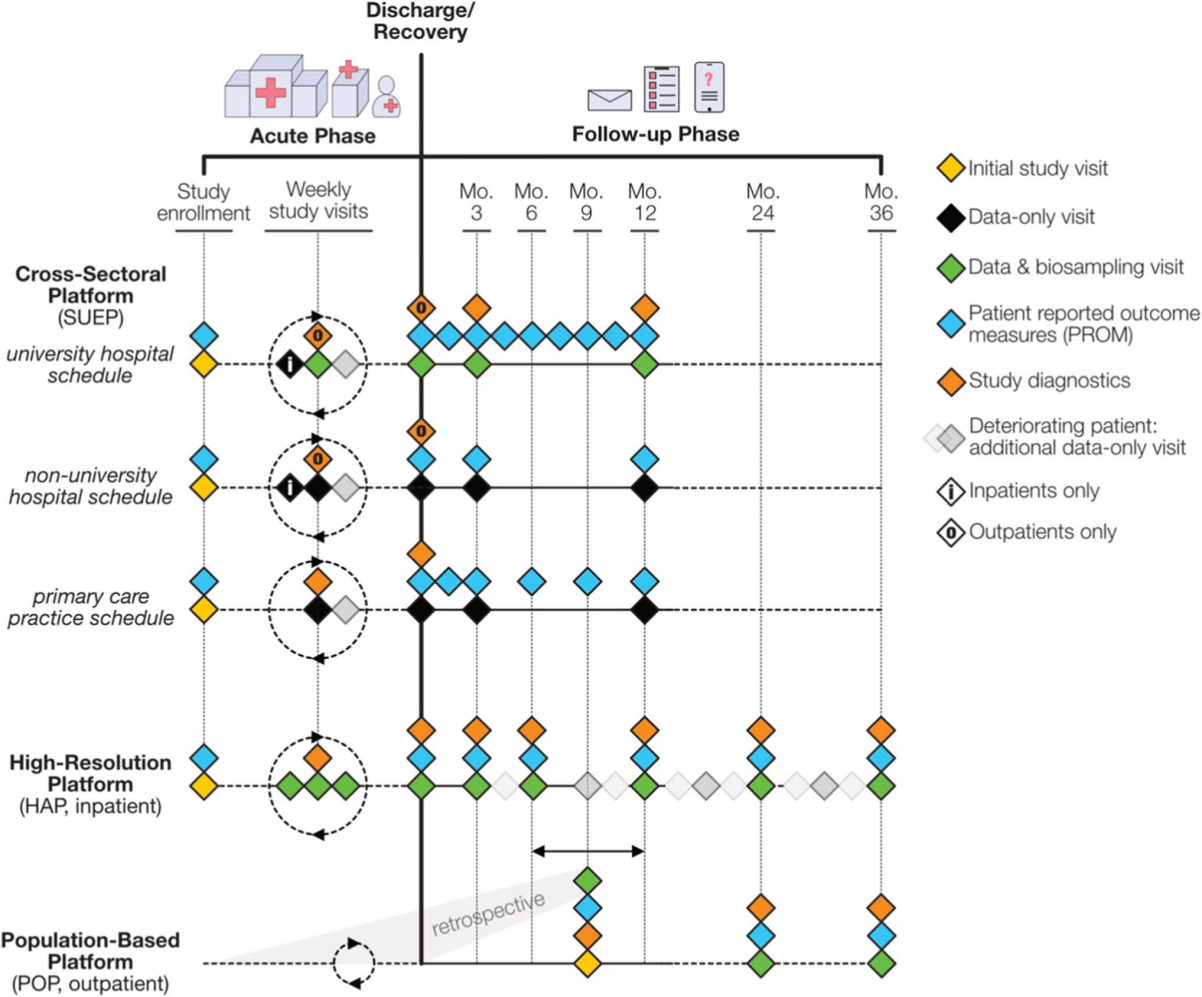
Visit schedules of the three NAPKON cohort platforms. During the acute phase, data collection and various study diagnostics are scheduled weekly. In case of complications, routine laboratory data and vitals parameters are additionally documented once a week. University hospitals collect biosamples weekly during study visits. Follow-up visits (scheduled in reference to initial diagnosis of SARS-CoV-2 infection) of patients include in-clinic study diagnostics (with biosampling at university hospitals) and questionnaires for PROMs. The POP documents the acute course of its patients retrospectively and performs its comprehensive in-clinic follow-up visits (including biosampling) roughly in yearly intervals [27].

The three NAPKON cohorts follow a harmonized visit schedule. Different intervals and visit types at the study center apply depending on the clinical setting (inpatient/outpatient), study site (university hospital, non-university hospital, primary care practice), patient age (adult vs. pediatric) and course of disease (acute vs. follow-up). Figure 1 juxtaposes the various study schedules of the three NAPKON cohorts. Recruitment of new patients for the SUEP and the HAP are planned until the end of 2023, for the POP until the end of 2022. Follow ups are planned until 2024 for all cohorts. Patients lost to follow-up are not replaced.

### Clinical assessment

During study and follow-up visits, patients undergo age adapted comprehensive clinical assessments according to the respective cohort protocol. All cohort platforms perform extensive laboratory testing, echocardiography, spirometry with full body plethysmography and diffusing capacity, vital sign measurement, and clinical examination. The HAP and the POP perform additional tests of fitness and organ function as well as imaging studies (see Table 2), such as brain magnetic resonance imaging (MRI) and cardio MRI examinations during convalescence, including, amongst others, quantitative multi parametric mapping [31, 32] and stress perfusion and late gadolinium enhancement [33].

### Biosample collection

Across the NAPKON cohorts, a common set of quality-assured biosamples is prospectively collected at specific study visits (see Fig. 1 for timing) and processed according to standard operating procedures (SOPs) for subsequent storage in local biobanks. Biosample metadata that fully captures all processing steps are stored in the cLIMS infrastructure to allow central tracking of all biosamples. Patients and/or their legal representatives can decline sampling at any time. Biosample collection primarily involves university sites but can also be conducted by non-university hospitals collaborating with local biobanks. The BCU coordinators train any local biobank staff via tele-education tools for all processes related to collection, processing, storage, and shipment of samples as described in the respective SOPs (recordings and documents available in German at www.bbmri.de/covid-19/nationales-pandemie-kohorten-netz/). Also, the BCU verifies compliance during regular audits at all sites. The NAPKON’s protocols permit study sites to collect further optional biosamples for the site’s own research interests. Systematic molecular phenotyping includes epigenetic, transcriptomic, proteomic, immunogenicity and metabolomic analyses of the patient samples as well as sequencing of all respiratory samples.

### Data collection

In all the NAPKON cohorts, study personnel manually transfer primary data and metadata stored in the patient’s health care record at the respective study site or acquired during the study to eCRFs. All data are captured prospectively, apart from anamnestic pre-infection data and the POP’s retrospective assessment of the acute phase. Local study staff collects PROMs as paper-/web-based questionnaires or conducts phone interviews. Quality measures of the clinical data include:

- Automatic predefined plausibility and completeness checks in the eCRFs
- Local (Review A) and central (Review B) quality assessment
- Random source data verification of 10%-20% of the cases.

To further improve quality, the ECU provides a methodological-epidemiological consultation platform on issues related to the planning, conduct, and analysis of the cohorts. Data quality reports on selected indicators, primary coding of core data, definition of plausibility ranges, SOPs, and statistical analysis planning including sample size reviews for use and access procedures are provided as part of this service.

Given the harmonized but individual data collection of the three NAPKON cohorts, each maintains its dedicated eCRF. All NAPKON eCRFs contain the German Corona Consensus Dataset (GECCO-83) [34], ensuring syntactic and semantic interoperability for a core dataset via international terminologies (e.g., International Statistical Classification of Diseases and Related Health Problems, 10th revision, German modification (ICD-10-GM) [35], Logical Observation Identifiers Names and Codes (LOINC) [36], the Anatomical Therapeutic Chemical Classification System (ATC) [37], Systematized Nomenclature of Medicine Clinical Terms (SNOMED CT) [38]) and defined Health Level 7 (HL7) standard Fast Healthcare Interoperability Resources (FHIR) profiles [39]. The cohorts selected additional data elements by incorporating international data sets (e.g., ISARIC [40]), already established German COVID-19 cohorts (e.g., LEOSS [41], Pa-COVID-19 [42]), and suggestions of scientists (see section “Governance”). By choice of design, the NAPKON’s clinical/imaging data set definitions and biosample panel allow adjustments and extensions via add-on modules. Tables 1 and 2 provide an overview of the data currently collected across the three NAPKON cohorts.

The later presented baseline characteristics of the current NAPKON cohort use descriptive summary statistics for all patients included across the three cohorts that passed Review A. All analyses have been performed with the statistics software R, version 4.0.2.

### Study organization

#### Governance and use and access

**Fig. 2:**
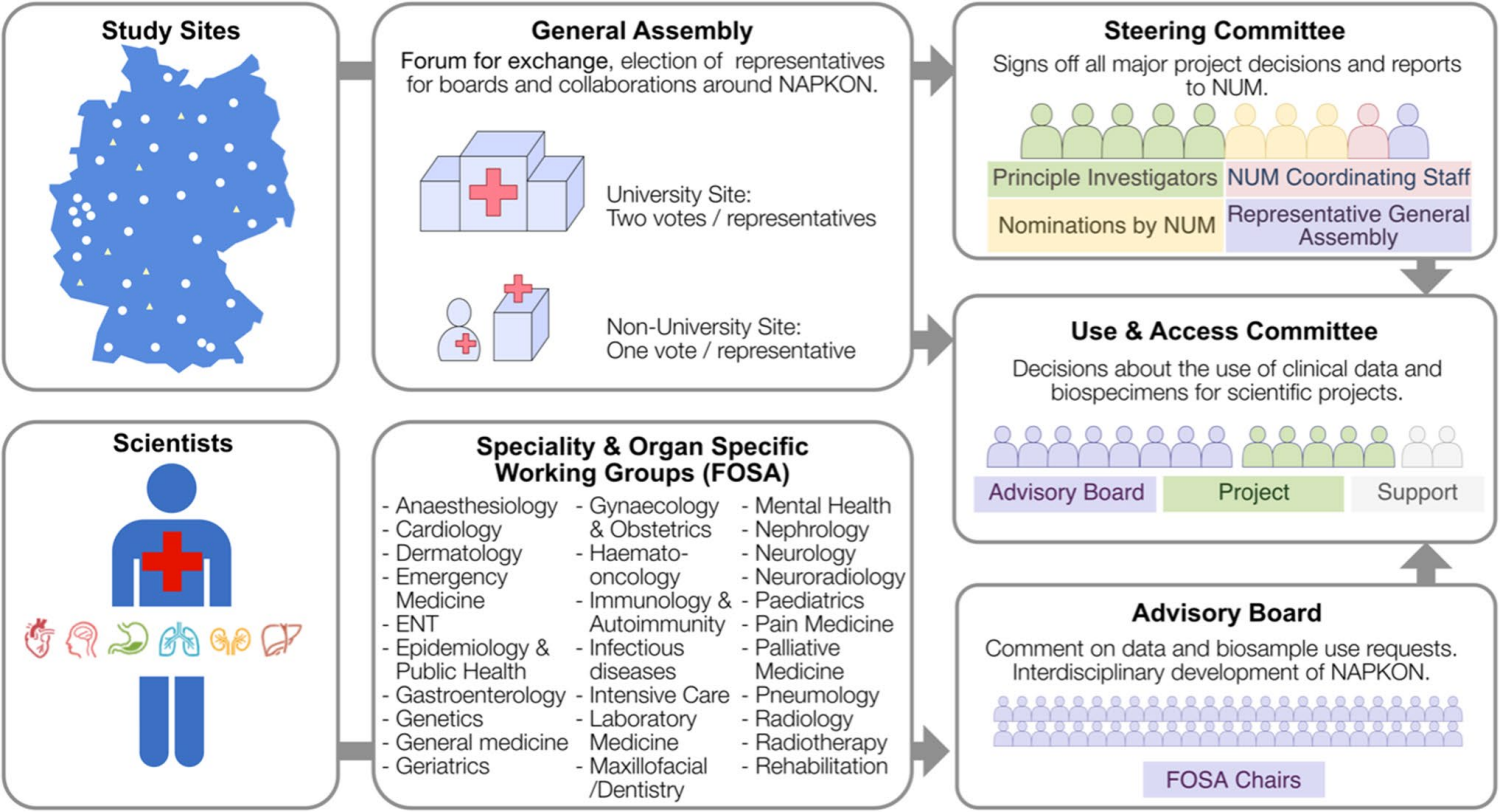
Flow-diagram of the NAPKON governance. Study sites and scientists are prominently included in most governance processes

The ICU assumes the overall coordination of the NAPKON. Figure 2 illustrates the stakeholders’ positions in the following governance structures: General Assembly (GA), Advisory Board (AB), Use & Access Committee (UAC), Steering Committee (SC), and Specialty- and Organ-Specific Working Groups (*Fach- und Organspezifische Arbeitsgruppen*, FOSAs). The SC devises and approves regulating documents for the SC, FOSA, and UAC, as well as the usage regulations and publication regulations. Rules of procedure require gender parity for the FOSAs, AB, and the SC and actively encourage it in the GA.

Groups greater than ten individuals from different university hospitals can establish a FOSA that is open to the general scientific community. The core responsibilities of the FOSAs are to comment on and revise the NAPKON’s data sets and develop subject-specific research questions for analyses. FOSA chairs form the AB, and interdisciplinary advise the NAPKON’s committees and study platforms.

The ICU hosts and supports regular meetings for all governance organs. Scientists can submit applications that outline the reasoning and methodology for their research questions and are also expected to describe how they plan to address limitations of the NAPKON data, e.g., when combining cases across different health care settings or recruitment funnels. Both, the Epidemiology Core Unit and the Advisory Board, participate in the UAC process to extend the UAC’s review. Data privacy & ethics

Study protocols and consent forms detail all patient-related activities for children, adolescents and adults in the NAPKON. The patient documents are harmonized in a way that enables the data from three cohorts to be used in an intersectional manner. All participants are informed about the shared data management and overarching governance and agree to the use of their data for research regarding the description, detection, treatment and prevention of SARS-CoV-2 infection and COVID-19 disease research via the consent form. Further information is provided on the patient information website, for example regarding responsibilities for data processing (https://napkon.de/pat/datenschutz). Patients and/or their legal representatives can withdraw their data or biosample use at any time without giving reasons (https://napkon.de/pat/datenschutz).

The DZHK Trusted Third Party provides centrally managed pseudonyms throughout the NAPKON which prevents identification of individuals by unauthorized persons while allowing linkage of different types of data and biosamples. Scientists working with the NAPKON data or biosamples must adhere to relevant UAC privacy regulations. A restrictive procedure for transmission of any data or biosamples to researchers who are not bound by European data privacy laws is in place. Members of the Use & Access process and individuals who manage incoming research proposals are subject to confidentiality agreements.

#### Project management infrastructure

The NAPKON self-hosts collaboration and project management solutions under the umbrella term “NAPKON Suite” on its website https://napkon.de. It includes collaborative cloud space, mailing lists, a contact directory, email inboxes, project management software, and administrative tools. Via a single-sign-on and group memberships, all participating scientists, local study teams, and governance members have access to respective services.

The NAPKON Suite provides supporting materials (e.g., SOPs, video recordings, FAQs, flow charts) to all participating study sites. The NAPKON’s homepage bundles all relevant information for different stakeholders, including patient information and short summaries of the UAC-approved scientific projects. Project milestones and deliverables are tracked in an OpenProject [43] implementation as part of the NAPKON Suite.

### Study budget

The total budget of the NAPKON from August 06, 2020 until December 31, 2024 is close to 55 Mio €, not including the funding for the DZHK. More than half of the total budget is used for case fees, about 15% site setup costs, with the remainder being allocated towards cohort governance and infrastructure core unit setup/management.

## Results

The results presented in the following reflect the status of the cohorts on April 01, 2022, roughly one and a half years after the first patient was recruited.

### Governance and use and access

The ICU set up all the NAPKON’s governance structures and reconciled legal documents with the NUM coordination (e.g., data privacy agreement, usage regulations, publication regulations, and others). The NAPKON’s SC convenes biweekly since September 08, 2020. The first digital GA meeting was held on December 17, 2020, the second on November 08, 2021. AB and UAC had their first meeting on February 04, 2021, and March 05, 2021, respectively. ICU held an additional study site & investigator meeting on February 28, 2021. Organized by the ICU, five events titled “FOSA Lectures” have taken place in which FOSAs shared and discussed their current findings on the COVID-19 pandemic.

By April 01, 2021, the NAPKON counts more than 1,500 involved members in the NAPKON Suite. About 70 of these members are part of the general coordination, who generally convene biweekly via video conferencing. Local study groups generally consist of about three to 20 people each. Twenty-eight different FOSAs were formed with over 600 national scientists (Fig. 2 lists all FOSA specialties). Consequently, the AB consists of 56 members. The NAPKON FOSAs were decision-maker in the content definition for three extension modules to the GECCO-83 developed within NAPKON to harmonize focused data collection in cardiology, pediatrics, and vaccination with respect to the pandemic development. Since January 01, 2022 the responsibilities of the FOSA and the AB have been extended across all NUM projects.

The NAPKON’s Use & Access process was tested in March 2021 and opened on April 26, 2021. It relies on the NUM usage regulations, the NAPKON/NUM publication regulations, and the NAPKON usage regulations for biosamples. Interested scientists can access those documents and submit research proposals via https://proskive.napkon.de. The UAC (see Fig. 2) discusses and votes on proposals while respecting efficient processes tailored to the current pandemic situation. Usage regulations grant government agencies and other public health institutions privileged access to the data collected in the NAPKON. The AB members can comment on incoming requests for data and biosamples. The first incoming applications were circulated in the AB on May 03, 2021, and in the UAC on May 12, 2021. By April 01, 2022, 92research proposals have been submitted to the UAC. Of those, the UAC approved 80, declined three with the remaining requests still pending. 24 proposals have received data already, three biosamples. Currently the average time from submission until approval is 12 days.

The ECU's methodological-epidemiological consultancy service has processed more than 20 scientific inquiries to date. ECU also reviews applications to the UAC regarding design and sample size. To describe the content of the cohort data, (center-specific) core analyses have been provided regularly since April 2021.

The integration core unit has developed concepts to incorporate data from cohorts collected prior to the NAPKON. Use cases are being implemented to demonstrate the feasibility of such data integration in the HAP and the SUEP.

### Cohorts and study population

All three NAPKON cohorts recruited their first patients in November 2020, 4 months after the initial submission of the NAPKON’s proposal to the NUM. For the SUEP, 28 university hospitals, 17 non-university hospitals, and 23 primary care practices recruit patients, for the HAP and the POP 10 and 3 university hospitals, respectively. 34 of 36 university hospitals in Germany participate in the NAPKON. The extensive protocols feature more than 90 SOPs for clinical tests and diagnostics, multiple imaging follow-ups, and standardized biosample collection. Twenty expressions of interest integrating previous studies’ data or biosamples into the NAPKON infrastructure were solicited by the IGCU, 13 of which met the criteria defined for successful integration. Two initial integration use cases for the HAP and the SUEP, respectively, were launched in April 2021 after a legal opinion helped clarify pending regulatory issues.

The FOSAs have thoroughly reviewed and updated the eCRFs and made many hundred individual suggestions, including about 150 new and 25 removed variables, the definition of plausibility limits, the creation of clinical definitions, the addition of anamnesis checklists, and the revision of the study concept for outpatient practices. The AB led and defined the PROM specification in the NAPKON and was heavily involved in the follow-up visit design. Dedicated committees have worked on add-on modules for cardiology, neurology, dermatology, and pediatrics. The pediatric module, includes specific case definitions for pediatric patients and controls, age-specific plausibility checks, age-adopted medical and social variables. It was activated on May 12, 2021. Other add-on modules (e.g., age-adopted PROMs) are expected to follow soon. The AB members further discussed ideas on maintaining the broadest possible interdisciplinary collaboration between the NUM and the NAPKON in the future. The ideas are part of the “NAPKON Follow-Up Application 2022–2024” that was submitted on June 30, 2021, to the NUM. The development of a generic recruitment infrastructure was finally included in the NUM Clinical Epidemiology and Study Platform (German: *NUM Klinische Epidemiologie und Studien Plattform*, NUKLEUS) project description, which was approved on December 14, 2021.

By April 01, 2022, the NAPKON has recruited a total of 5298 patients across all cohorts including 17 controls in the SUEP. Study sites have recruited almost 600 patients per month at peak times. Table 4 presents the characteristics of the study population in the style of the ECU’s core analyses for 4727/5298 (89%) SARS-CoV-2 positive patients with a Review A status (local data quality review performed). 2202/4727 (47%) are female, 2524/4727 (53%) are male, 0 are non-binary. The median age was 56 (IQR 42–68), 57 (IQR 47–65), and 46 (IQR 31–68) for the SUEP, the HAP, and the POP respectively. 50 pediatric cases had a positive Review A status at the point of the analysis. 1834/4161 (44%; SUEP: 88%, HAP: 100%, POP: 7%) patients were hospitalized, 611/3997 (15%; SUEP: 33%, HAP: 38%, POP: 2%) admitted to an intensive care unit, and 214/1800 (4%; SUEP: 12%, HAP: 12%, POP: 0%) patients deceased while enrolled. 1741/3581 (49%; SUEP: 49%; HAP: 19%; POP: 55%) had a full vaccination status. 696/1258 (55%), 113/182 (62%), and 256/694 (37%) patients have been followed-up at 3, 6 and 12 months across the cohorts. The POP did baseline visits with 2,346 patients at 6-12 months post primary infection. Pre-existing comorbidity distributions for the SUEP, the HAP and the POP included 50%, 50%, 29% chronic cardiovascular disease; 19%, 21%, and 19% chronic lung disease; 12%, 17%, and 0.5% chronic kidney disease; 13%, 15%, and 25% chronic neurological or psychiatric disease. 130/5,298 (2%) patients withdrew at least parts of their data by March 2022. No information about the completeness of recruitment per center was available at the point of analysis. Missing data due to a pending Review A status or other reasons is indicated in Table 4.

**Table 4:** Description of the study population with review A status (with a total patient population of 5298) by cohort until April 01, 2022

Variable	N1	Statistic	HAP, N = 544	POP, N = 2346	SÜP, N = 1837
Age (numeric)	4727	Median (IQR)	57 (47, 65)	46 (31, 57)	56 (42, 68)
Age (categorical)	4727				
< 18		n (%)	0 (0%)	0 (0%)	50 (2.7%)
18–29		n (%)	33 (6.1%)	501 (21%)	138 (7.5%)
30–39		n (%)	51 (9.4%)	446 (19%)	205 (11%)
40–49		n (%)	83 (15%)	360 (15%)	280 (15%)
50–59		n (%)	156 (29%)	608 (26%)	368 (20%)
60–69		n (%)	128 (24%)	260 (11%)	379 (21%)
70–79		n (%)	69 (13%)	140 (6.0%)	255 (14%)
80+		n (%)	24 (4.4%)	31 (1.3%)	162 (8.8%)
Gender	4726				
Female		n (%)	174 (32%)	1305 (56%)	723 (39%)
Male		n (%)	370 (68%)	1040 (44%)	1114 (61%)
Non-binary		n (%)	0 (0%)	0 (0%)	0 (0%)
Missing or review A pending		n	0	1	0
Smoking (past or current smoker)	3867				
Yes		n (%)	28 (6.8%)	1068 (49%)	128 (9.9%)
No		n (%)	381 (93%)	1100 (51%)	1162 (90%)
Missing or review A pending		n	135	178	547
Alcohol	2433				
Never		n (%)	0 (NA%)	183 (13%)	516 (50%)
Up to 4 times monthly		n (%)	0 (NA%)	676 (48%)	377 (36%)
Multiple times weekly		n (%)	0 (NA%)	536 (38%)	145 (14%)
Missing or review A pending		n	544	951	799
Obesity at inclusion (BMI ≥ 30 kg/m^2^)	4169				
No		n (%)	311 (65%)	1758 (76%)	900 (66%)
Yes		n (%)	167 (35%)	566 (24%)	467 (34%)
Missing or review A pending		n	66	22	470
SARS-CoV-2 vaccined	3581				
Yes		n (%)	93 (19%)	1189 (55%)	459 (49%)
No		n (%)	393 (81%)	965 (45%)	482 (51%)
Missing or review A pending		n	58	192	896
In-patient ever	4161				
Yes		n (%)	544 (100%)	170 (7.3%)	1120 (88%)
No		n (%)	0 (0%)	2172 (93%)	155 (12%)
Missing or review A pending		n	0	4	562
Intensive stay ever	3997				
Yes		n (%)	208 (38%)	36 (1.5%)	367 (33%)
No		n (%)	336 (62%)	2301 (98%)	749 (67%)
Missing or review A pending		n	0	9	721
Covid-associated oxygenation	4619				
Invasive/non-invasive ventilation		n (%)	141 (26%)	17 (0.7%)	299 (17%)
O2-therapy only		n (%)	260 (48%)	93 (4.0%)	746 (43%)
No assistance		n (%)	142 (26%)	2222 (95%)	699 (40%)
Missing or review A pending		n	1	14	93
Extracorporeal membrane oxygenation (ECMO)	4047				
Yes		n (%)	60 (13%)	1 (<0.1%)	47 (3.8%)
No		n (%)	417 (87%)	2334 (100%)	1188 (96%)
Missing or review A pending		n	67	11	602
Chronic cardiovascular disease	3842				
Yes		n (%)	269 (50%)	601 (29%)	622 (50%)
No		n (%)	265 (50%)	1456 (71%)	629 (50%)
Missing or review A pending		n	10	289	586
Chronic lung disease	4013				
Yes		n (%)	109 (21%)	425 (19%)	235 (19%)
No		n (%)	417 (79%)	1822 (81%)	1005 (81%)
Missing or review A pending		n	18	99	597
Chronic kidney disease	4088				
Yes		n (%)	88 (17%)	8 (0.3%)	142 (12%)
No		n (%)	436 (83%)	2323 (100%)	1091 (88%)
Missing or review A pending		n	20	15	604
Chronic liver disease	3722				
Yes		n (%)	44 (8.4%)	181 (9.2%)	84 (6.8%)
No		n (%)	480 (92%)	1788 (91%)	1145 (93%)
Missing or Review A pending		n	20	377	608
Rheumatological/immunological disease	4053				
Yes		n (%)	32 (6.1%)	219 (9.5%)	60 (4.9%)
No		n (%)	492 (94%)	2075 (90%)	1175 (95%)
Missing or review A pending		n	20	52	602
Diabetes mellitus	4001				
Yes		n (%)	110 (21%)	101 (4.5%)	266 (21%)
No		n (%)	418 (79%)	2129 (95%)	977 (79%)
Missing or review A pending		n	16	116	594
Solid tumor disease	4092				
Yes		n (%)	57 (11%)	39 (1.7%)	150 (12%)
No		n (%)	478 (89%)	2294 (98%)	1074 (88%)
Missing or review A pending		n	9	13	613
Haematological-oncological disease	4074				
Yes		n (%)	29 (5.5%)	7 (0.3%)	63 (5.2%)
No		n (%)	498 (94%)	2323 (100%)	1154 (95%)
Missing or review A pending		n	17	16	620
HIV infection	3948				
Yes		n (%)	3 (0.6%)	2 (<0.1%)	17 (1.5%)
No		n (%)	473 (99%)	2336 (100%)	1117 (99%)
Missing or review A pending		n	68	8	703
Chronic neurological or psychiatric disease	3997				
Yes		n (%)	81 (15%)	570 (25%)	159 (13%)
No		n (%)	451 (85%)	1690 (75%)	1046 (87%)
Missing or review A pending		n	12	86	632
History of organ transplantation	4106				
Yes		n (%)	56 (10%)	8 (0.3%)	60 (4.9%)
No		n (%)	479 (90%)	2327 (100%)	1176 (95%)
Missing or review A pending		n	9	11	601
General symptoms	3711				
Yes		n (%)	246 (59%)	1964 (95%)	979 (80%)
No		n (%)	172 (41%)	110 (5.3%)	240 (20%)
Missing or Review A pending		n	126	272	618
Respiratory symptoms	3726				
Yes		n (%)	253 (61%)	1974 (95%)	979 (80%)
No		n (%)	165 (39%)	110 (5.3%)	245 (20%)
Missing or review A pending		n	126	262	613
Gastrointestinal symptoms	2626				
Yes		n (%)	93 (22%)	900 (89%)	448 (37%)
No		n (%)	325 (78%)	110 (11%)	750 (63%)
Missing or review A pending		n	126	1336	639
Neurological symptoms	2829				
Yes		n (%)	94 (22%)	1094 (91%)	424 (35%)
No		n (%)	324 (78%)	110 (9.1%)	783 (65%)
Missing or review A pending		n	126	1142	630
Other symptoms	2750				
Yes		n (%)	93 (22%)	1019 (90%)	387 (32%)
No		n (%)	325 (78%)	110 (9.7%)	816 (68%)
Missing or review A pending		n	126	1217	634
Asymptomatic	4012				
Yes		n (%)	4 (0.8%)	110 (4.9%)	62 (5.0%)
No		n (%)	520 (99%)	2134 (95%)	1182 (95%)
Missing or review A pending		n	20	102	593
Early outcome	1800				
Discharged home/ambulatory care		n (%)	413 (79%)	0 (NA%)	834 (65%)
Unknown or no change yet		n (%)	0 (0%)	0 (NA%)	185 (14%)
Transferred to or from another facility		n (%)	47 (9.0%)	0 (NA%)	107 (8.4%)
Deceased		n (%)	62 (12%)	0 (NA%)	152 (12%)
Missing or review A pending		n	22	2346	559
3M follow-up conducted	1258				
Yes		n (%)	162 (69%)	0 (NA%)	534 (52%)
No		n (%)	74 (31%)	0 (NA%)	488 (48%)
Missing or review A pending		n	308	2346	815
6M follow-up conducted	182				
Yes		n (%)	113 (62%)	0 (NA%)	0 (NA%)
No		n (%)	69 (38%)	0 (NA%)	0 (NA%)
Missing or review A pending		n	362	2346	1837
12M follow-up conducted	694				
Yes		n (%)	37 (35%)	0 (NA%)	219 (37%)
No		n (%)	68 (65%)	0 (NA%)	370 (63%)
Missing or review A pending		n	439	2346	1248

SUEP, cross-sectoral platform; HAP, high-resolution platform; POP, population-based platform

Baseline characteristics for the SUEP and the HAP correspond to the baseline visit during acute infection, for the POP to the first baseline visit 6-12 months after infection

### Biosamples

As of April 03, 2022, 4349/5298 (82%) patients had at least one study visit with biosampling, 1442 in the SUEP, 439 in the HAP, and 2,468 in the POP. On average, a patient had 2 study visits with biosampling. This totals in 8845 biosample panels stored in 34 local biobanks (see Table 5 for details). Follow-up samples for month 3, 6, 12, and 24 exist from 740, 1942, 209, and 29 patients, respectively. The BCU performed 34 audits with 79 deviations and 212 recommendations by April 01, 2022.

**Table 5:** Collected number of respective biosamples until April 03, 2022.

		Total	SUEP	HAP	POP
Patients with biosamples		4349	1442	439	2469
Visits with biosampling		8845	3915	2445	2485
Follow-up visits total		2920	754	346	1822
3 months		740	583	155	2
6 months		1942	3	120	1819
12 months		209	166	43	0
24 months		29	0	28	1
Average visit with biosampling per patient		2	3	6	1
	*Intended use*				
EDTA blood	Plasma: proteome, metabolome, biomarker analysis; DNA: genome, epigenome	10,988	3974	2470	4544
Serum	Clinical and biomarker analysis	8636	3760	2435	2443
Respiratory sample^a^	Determination of virus subtype, microbiome	7077	3644	950	2483
Oro/nasopharyngeal swab^a^		2916	2358	468	90
Saliva^a^		4091	1217	482	2392
ENTA^a,b^		65	64	0	1
BAL^a,b^		5	5	0	0
PAXgene RNA	Transcriptome	8362	3627	2407	2328
Citrate blood	Analysis of coagulation factors, biomarkers	10,727	4187	2470	4070
PBMC (all variants)	Analysis of cellular immune response	11,484	4701	4361	2422
CPT		5918	3337	936	1645
EDTA		1139	632	507	0
Heparine		4427	732	2918	777
Urine^a^	Metabolome, kidney measures	6358	3055	959	2344

BAL, Bronchoalveolar lavage; ENTA, endotracheal aspiration; PBMC, peripheral blood mononuclear cells; CPT, cell preparation tube; SUEP, cross-sectoral platform; HAP, high-resolution platform; POP, population-based platform

^a^Only one sample taken per week

^b^Only for intensive care patients and clinical indication

## Discussion

During the COVID-19 pandemic, many countries pooled relevant health care data on thousands to millions of COVID-19 cases (i.e., United States [15, 44, 45], Scotland [17], United Kingdom [16, 46], Canada [24], China [23], Iran [20], Qatar [21], Middle East [22], Mexico [19] or South Korea [18]). Although dedicated national COVID-19 biobank activities started as early as February 2020 [47] and many large national multi-center observational studies collecting data and biosamples for selected conditions exist [48, 49], the number of national prospective studies following an interdisciplinary approach similar to the NAPKON is still small. This is not particularly surprising, as the pandemic impacted the biobanking activities globally [50], and the multi-site roll-out of interdisciplinary study protocols is exceptionally resource-intensive. Currently, the three comparable studies to the NAPKON approach are Canada’s CANCOV [51], Brazil’s SARS-Brazil [52] and France’s FrenchCOVID [14], targeting longitudinal, multicenter data and biosamples of 2000, 2000, and 5000 patients, respectively.

Launched in February 2020, FrenchCOVID assesses clinical features and pathogen evolution of SARS-CoV-2 infected inpatients daily for 15 days, then weekly up to 100 days, and invites patients for follow-up visits at 3 and 6 months. In addition to clinical data, the study collects biosamples (including blood, urine, stool, respiratory samples, samples from infected sites, and cerebrospinal fluid) of patients of any age. Study sites perform no additional clinical examinations or diagnostics. Recruitment happens at hospitals only (81 sites in total), including many university sites [14]. SARS-Brazil already enrolled more than 1500 of the initially planned 2000 hospitalized adult SARS-CoV-2 infected patients. Biosamples include blood, serum, plasma, and nasal swabs, with a 60 day observational period after inclusion [52]. CANCOV follows a stratified recruitment approach similar to the NAPKON since April 2020, including out- and inpatients of varying disease severity. Thirty-two sites are involved. Its visit schedule collects data and biosamples at baseline, day 7, day 30, 3, 6 and 12 months, including quality of life measures and additional physical examinations [51].

In this context, we highlight several strengths of the NAPKON. The NAPKON overcomes limitations of previous German cohorts (e.g., anonymous recruitment without follow-ups in LEOSS, single-center collection in Pa-COVID-19) and its multi-layered cohort recruitment strategy covers the full SARS-CoV-2 spectrum across all ages, disease severities, and health care sectors. Differentiators include the extensive biosample collection, inclusion of pediatric patients, and collaboration with local health authorities for representative sampling. The visit schedule includes adaptive acute (e.g., continued weekly visits during hospitalization) and detailed follow-up (e.g., continued PROMs and follow-ups up to 3 years) elements in addition to comprehensive study diagnostics. The NAPKON is well equipped to validate previous findings [53, 54], focus on neglected nuances, add to the understanding of new variants of concern [55], and the future effects anticipated from PCS [56].

The most relevant limitations and challenges of the NAPKON include a relatively small number of non-university study sites and unsatisfactory linkage to electronic health care records. Also, generalizability to other less resourced health care systems and non-European-ancestry populations will probably be limited. The deployment of documentation staff allows for far-reaching data collection across IT systems, but sole reliance on manual data transfer is error-prone and cost-intensive. The NAPKON’s extensive infrastructure had to be established in an ongoing pandemic context; thus, it had a delayed start compared to international cohorts, missing out on notable opportunities in the first wave in 2020 (e.g., early contributions to the understanding of diagnostics, pathophysiology, virus subtypes and the treatment). Via the activities of the IGCU this may partly be compensated for. We were not able to compile data on completeness of recruitment/response rates for this article and hope to provide these in upcoming individual in-depth cohort descriptions.

NAPKON has already been remarkably successful although the pace of its development and the circumstances have been challenging. Data and biosamples are heavily requested and the NAPKON established collaborations with consortia such as *Connecting European Cohorts to Increase Common and Effective Response to SARS-CoV-2 Pandemic* (ORCHESTRA) [57]. With the NAPKON, we established a sustainable and open clinical research network across Germany that through continuous and interdisciplinary development is determined to become a core infrastructure for prospective, interventional clinical research in a consolidated NUM. Complemented by current preparations towards a NAPKON clinical trial platform inspired by the vastly successful RECOVERY study in the United Kingdom [58], this will also allow and expedite conduct of phase II/III clinical trials within the network.. While these infrastructures create opportunity for virtually all major fields of medical research that require such large-scale effort, they create preparedness for handling WHO’s list of priority diseases [59] or novel pathogens of natural or artificial origin.

## Supplementary Information

Below is the link to the electronic supplementary material.

Supplementary file1 (DOCX 14 kb)

## Data Availability

Data is available under the regulations of NAPKON’s Use & Access process regulations (https://proskive.napkon.de).

## Code availability

The code of the analysis or NAPKON Suite portal is available from corresponding author upon reasonable request.
