# Supplementary material for: The German National Pandemic Cohort Network (NAPKON): rationale, study design and baseline characteristics
Source: Eur J Epidemiol. 2022 Jul 29;37(8):849–70. doi: 10.1007/s10654-022-00896-z (PMC9336157; doi:10.1007/s10654-022-00896-z)
Supplement: Supplementary file 1 — Supplementary file1 (DOCX 14 kb) [file 10654_2022_896_MOESM1_ESM.docx]

| **Table S1:** Strata for recruitment by patient population and health care facility. At total of 7,000 patients are planned to be recruited and followed up by the end of 2024.   \|  \| University Hospitals \| Non-university hospital and primary care practices \| Post-SARS-CoV-2 infection (outpatient) \| \| --- \| --- \| --- \| --- \| \| Asymptomatic and mildly ill \| 10% \| 4% \| 30% \| \| Severely ill (e.g., hospitalized) \| 12.5% \| 4.5% \| 7.5% \| \| Critically ill (e.g., intensive care unit) \| 10% \| 4% \| 2.5% \| \| **Total SARS-CoV-2 infected patient population** \| **32.5%** \| **12.5%** \| **40%** \| \| Control group \| 7.5% \| 2.5% \| 5% \| \| **Total study population** \| **40%** \| **15%** \| **45%** \| |
| --- | --- | --- | --- | --- | --- | --- | --- | --- | --- | --- | --- | --- | --- | --- | --- | --- | --- | --- | --- | --- | --- | --- | --- | --- | --- | --- | --- | --- |
